# Knee Acoustic Emissions as a Digital Biomarker of Disease Status in Juvenile Idiopathic Arthritis

**DOI:** 10.3389/fdgth.2020.571839

**Published:** 2020-11-19

**Authors:** Daniel C. Whittingslow, Jonathan Zia, Sevda Gharehbaghi, Talia Gergely, Lori A. Ponder, Sampath Prahalad, Omer T. Inan

**Affiliations:** ^1^Coulter Department of Biomedical Engineering, Georgia Institute of Technology, Emory University, Atlanta, GA, United States; ^2^Emory University School of Medicine, Atlanta, GA, United States; ^3^Department of Electrical and Computer Engineering, Georgia Institute of Technology, Atlanta, GA, United States

**Keywords:** wearable sensors, machine learning, juvenile idiopathic arthiritis, acoustic sensing, signal processing

## Abstract

In this paper, we quantify the joint acoustic emissions (JAEs) from the knees of children with juvenile idiopathic arthritis (JIA) and support their use as a novel biomarker of the disease. JIA is the most common rheumatic disease of childhood; it has a highly variable presentation, and few reliable biomarkers which makes diagnosis and personalization of care difficult. The knee is the most commonly affected joint with hallmark synovitis and inflammation that can extend to damage the underlying cartilage and bone. During movement of the knee, internal friction creates JAEs that can be non-invasively measured. We hypothesize that these JAEs contain clinically relevant information that could be used for the diagnosis and personalization of treatment of JIA. In this study, we record and compare the JAEs from 25 patients with JIA−10 of whom were recorded a second time 3–6 months later—and 18 healthy age- and sex-matched controls. We compute signal features from each of those record cycles of flexion/extension and train a logistic regression classification model. The model classified each cycle as having JIA or being healthy with 84.4% accuracy using leave-one-subject-out cross validation (LOSO-CV). When assessing the full JAE recording of a subject (which contained at least 8 cycles of flexion/extension), a majority vote of the cycle labels accurately classified the subjects as having JIA or being healthy 100% of the time. Using the output probabilities of a JIA class as a basis for a joint health score and test it on the follow-up patient recordings. In all 10 of our 6-week follow-up recordings, the score accurately tracked with successful treatment of the condition. Our proposed JAE-based classification model of JIA presents a compelling case for incorporating this novel joint health assessment technique into the clinical work-up and monitoring of JIA.

## Introduction

Juvenile idiopathic arthritis (JIA) describes a heterogeneous group of arthritides that present in children. JIA encompasses all forms of arthritis that begin before a patient is 16 years old, lasts for at least 6 weeks, and are of an unknown origin. It is a leading cause of disability and the most common chronic rheumatic disease of childhood with a prevalence of 150 cases per 100,000 (1). It is an autoimmune disorder with a complex etiology thought to be related to a combination of pre-disposing genetic factors and environmental influence (2, 3).

The heterogeneity of presentation sometimes makes diagnosing JIA difficult. This difficulty is exacerbated by the lack of conclusive, diagnostic laboratory tests. Diagnosis currently relies on taking a thorough history, physical exam, and several laboratory and imaging studies (4). Once diagnosed, to select the most suitable treatment for JIA, the disease should be classified into its subtype. JIA is divided into seven subtypes based on laboratory and clinically observed features (5, 6). To determine the most appropriate subtype, and thus the most effective therapy, an extensive workup must be performed on each patient. This is a time and resource intensive process. These workups include the history and physical exam, as well as a full blood exam. Imaging studies are also commonly used to grade the disease. After diagnosis, the goal is to enable the child to resume normal childhood activities with normal growth and development (4). Managing JIA requires a combination of pharmacological interventions, physical and occupational therapy, and psychosocial support. The pharmacological treatment may involve corticosteroids, non-steroidal anti-inflammatory drugs (NSAIDS), or disease-modifying anti-rheumatic drugs (DMARDs) including biological response modifiers (7–9). This treatment protocol is largely reactive with decisions made based on subjective and qualitative measures of response to therapy.

Early diagnosis with effective treatment is necessary for preventing the long-term sequela of JIA (4). However, JIA's highly variable presentation, symptomatology and course make diagnosis and selection of the most suitable treatment difficult. Pediatric rheumatologists are most well-suited for diagnosing and treating JIA; however, there is currently a severe shortage of pediatric rheumatologists. As of 2019, there are fewer than 400 board-certified and practicing pediatric rheumatologists in the United States. This shortage contributes to only one in four children with JIA being able to regularly see a pediatric rheumatologist (10, 11). To address the difficulty of diagnosis, subjectivity of treatment, and severe lack of access to pediatric rheumatologists, more research must be performed in to develop objective biomarkers of JIA. A suitable biomarker could help more effectively diagnose patients, identify risk profiles, and predict/track an individual's response to treatment. Additionally, the development of such a biomarker could allow for more effective translation of the many genetic and immunological mechanistic studies of the disease to further improve clinical outcomes. Ideally, this biomarker would also be readily measurable with affordable technologies, so that JIA could be easily diagnosed and monitored by non-specialist healthcare workers.

The use of acoustics—recording the sounds that the joints make during movement—could provide a basis for developing such a biomarker (12). These sounds, or joint acoustic emissions (JAEs), can be readily measured on the surface of the skin and have shown promise in diagnosing joint pathologies and injuries. Most existing research into JAEs has focused on developing diagnostic techniques to differentiate “healthy” vs. “unhealthy” joints (13, 14). In one study, osteoarthritic knees were found to produce more frequent, louder, and longer duration acoustic emissions when compared against healthy knees (15). In the case of a chronic condition—such as JIA—JAEs could serve as a means of not only diagnosing but also longitudinally monitoring the conditioning. If JAEs show a correlation with disease status in JIA, they could regularly be measured to help personalize the management of JIA. Until recently, longitudinal assessment using JAEs in healthcare was not feasible due to a lack of technologies for recording JAEs outside of a laboratory or clinical setting. However, the development and application of piezoelectric accelerometers to JAE assessment has substantially advanced the field. This type of sensor is sensitive to physical vibrations (such as those seen on the skin during joint articulation), but does not substantially record external noises (16). JAE assessment technologies if properly applied to JIA, could lead to earlier diagnosis, improved and personalized care, and could serve as an objective measure in the next generation of clinical trials.

**Figure 1:**
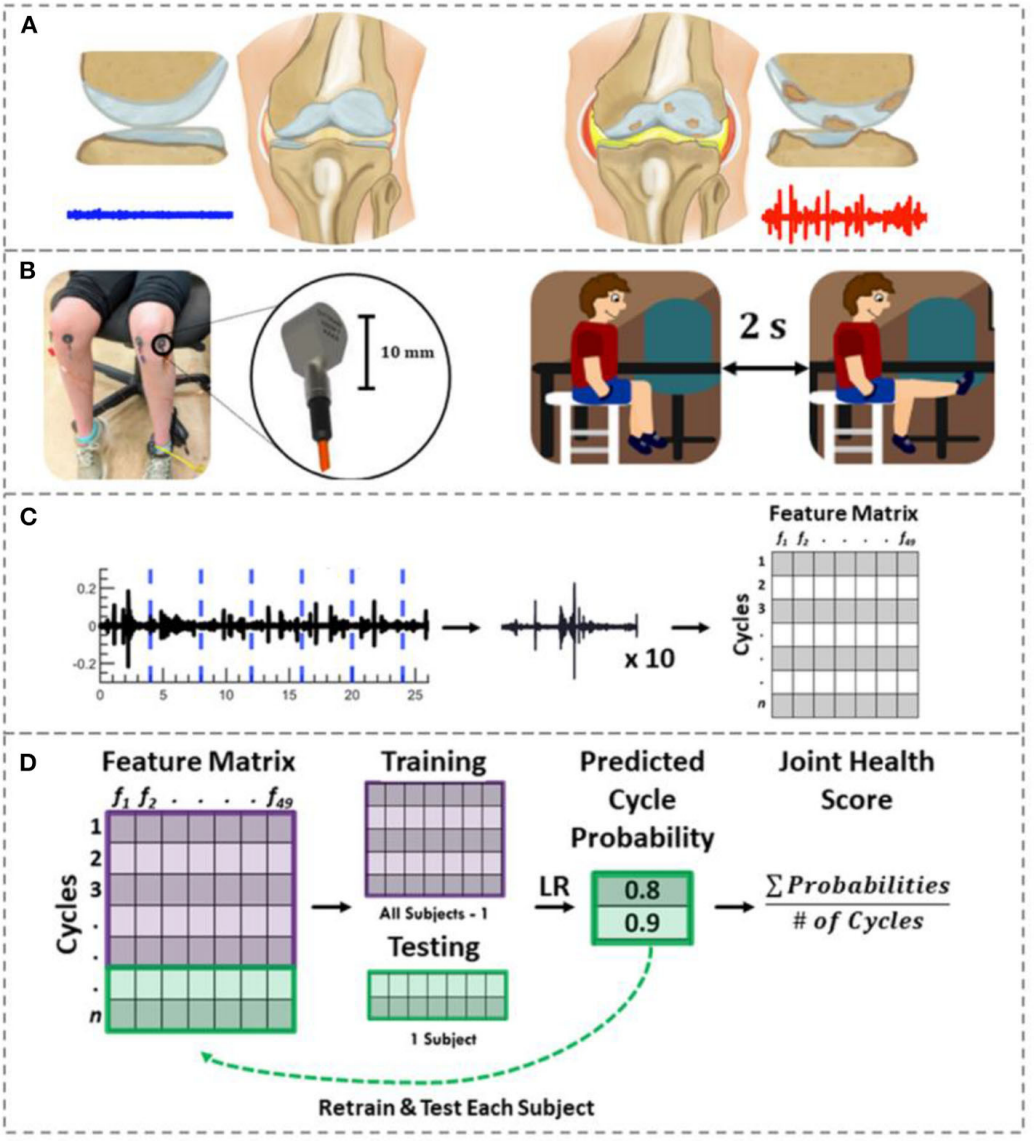
Joint acoustic emission overview. **(A)** A healthy knee articulates smoothly due to its smooth cartilage and appropriate amount/constituency of synovial fluid. This smooth articulation creates a noise-like JAE (blue). In JIA, thickened/inflamed synovium with excessive joint effusions, cartilage loss and/or bone erosions may be observed. These changes are hypothesized to create a JAE with several large spikes (red). **(B)** To record the knee JAEs, two contact accelerometers were placed on each child's knees. They viewed and replicated the movements in an instructional cartoon during JAE recording such that their movement speed and range of motion was controlled. **(C)** The resulting JAEs were split into their approximately ten component cycles. Forty-nine features were calculated to describe these cycles. The features, subject numbers, and clinically determined disease status were fit to a feature matrix. **(D)** Using logistic regression and LOSO-CV, the probability of each cycle belonging to JIA were calculated. The average of those cycle probabilities is used as a “joint health score” to indicate the severity of JIA. If the majority of cycles for a given subject had a probability of JIA ≥ 0.5, that subject was classified as having JIA.

In this paper, we explore the potential of using JAE analysis to diagnose and longitudinally track JIA. In this work, JAEs were recorded from the knees - one of the most commonly affected joints in JIA (17, 18). Our team recently showed that by damaging the meniscus in a cadaver model of the knee, the resulting JAEs were substantially altered (19). In the case of JIA, affected joints are characterized by persistent joint swelling caused by an accumulation of synovial fluid and thickening of the synovial lining (3) (Figure 1A). We hypothesize that these pathologic changes in the knee will similarly alter JAE profile of the knee. If that hypothesis is supported, the JAEs of the knee could then be correlated with disease status.

To test this hypothesis, first, we built a custom hardware and software setup for recording JAEs and designed a novel signal analysis algorithm that windows the JAE recording based on the cycles of flexion/extension (Figures 1B–D). We placed two piezoelectric accelerometers medial and lateral to the distal patellar tendon, and an inertial measurement unit (IMU) around the ankle. With the hardware in place, the subject performs 10 flexion/extension cycles. The JAEs from the knees of two groups of children are recorded: one group had active JIA and the other was an age- and sex-matched healthy control group. To assess the effectiveness of JAEs for tracking therapeutic efficacy and changes in disease status, we also recorded the JAEs from the children with JIA 6 weeks after successful treatment. Our proposed algorithm, powered by logistic regression, analyses 49 signal features (summarized in Supplementary Table 1) of each individual cycle of flexion/extension and outputs the probability that a cycle belongs to a patient with JIA. This output probability forms the basis for our proposed JIA digital biomarker. Finally, we assess the importance of each signal feature in the algorithm as well as the accuracy and generalizability of the model using leave-one-subject-out cross-validation (LOSO-CV).

## Results

### Qualitative Comparison of Knee JAEs

**Figure 2:**
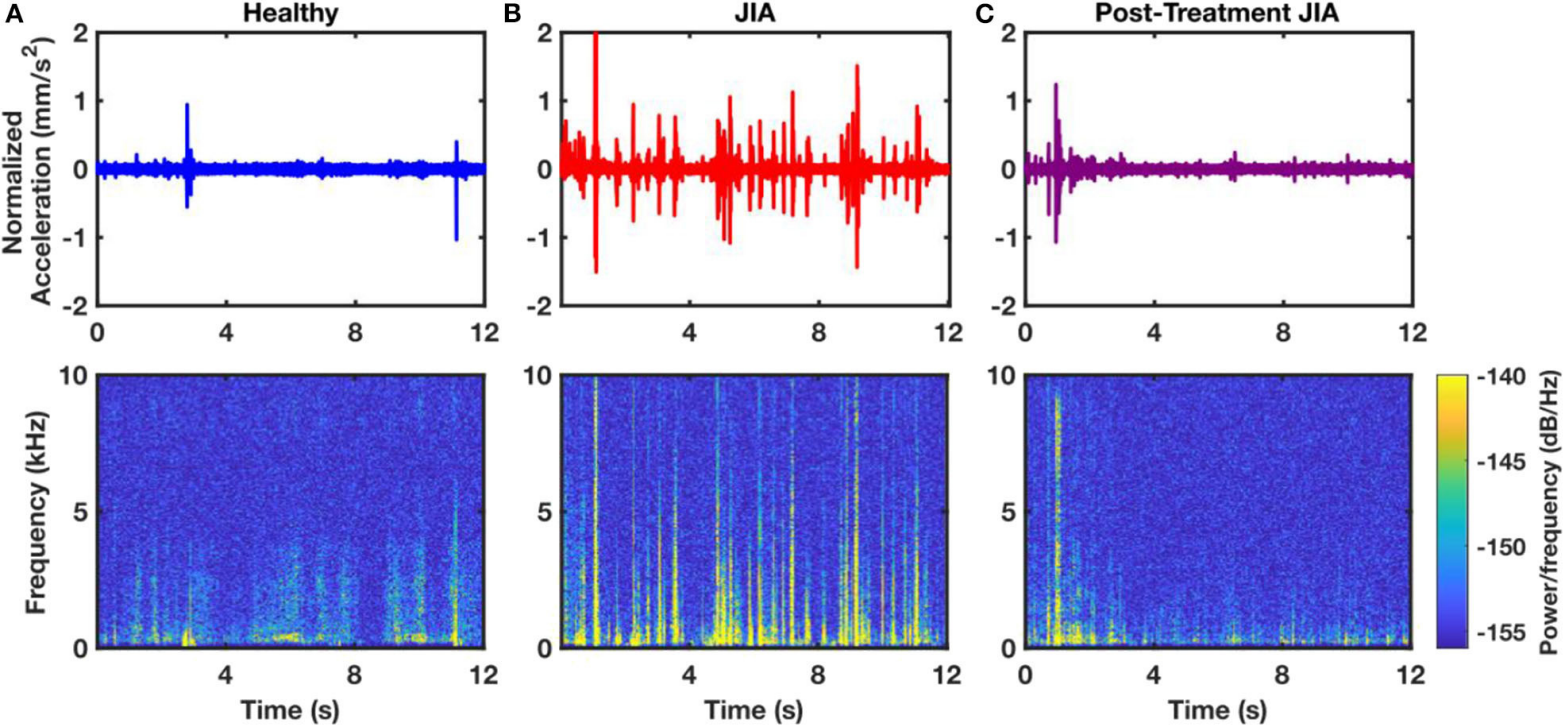
Representative time-domain and spectrogram plots of JAEs from a sample healthy control **(A)**, a subject with active JIA **(B)**, and that same subject after 6-weeks of successful treatment **(C)**. The 12 s of the JAEs represent approximately 4 flexion/extension cycles. Spectrogram of the subject with JIA contains more high power and high frequency components compared to that of healthy and post-treatment subjects.

The JAEs were recorded from the knees of two groups of children. One group had actively inflamed knees with either newly diagnosed or poorly controlled JIA as diagnosed by their treating pediatric rheumatologist; the other group was composed of age- and sex-matched health controls with no JIA or known injuries to the knee. There are several notable differences in the time-domain patterns of the JAEs between these groups. The 18 healthy controls had no noticeable peaks in their audio signals and upon listening the recorded JAEs resembled white noise (Figure 2A). The 25 subjects with JIA consistently exhibited periodic, high-energy clicks in each flexion-extension cycle. These “clicks” have a spike-like appearance in the time-domain plot which correspond to the high power content in the higher frequency components in spectrogram (Figure 2B). Ten of these patients with JIA had a second recording after 6 weeks of treatment as prescribed by their treating pediatric rheumatologist. The JAEs of this follow-up group showed a large reduction in the amplitude and frequency of the clicks noted during their actively inflamed stage (Figure 2C). The post-treatment JAEs more closely resembled the healthy controls both in the time-domain and spectrogram plots of the JAEs as well as in audibly listening to the recordings. A representative subject's JAE recording from each of these groups is presented in Figure 2.

### Knee Audio Score Classification

**Figure 3:**
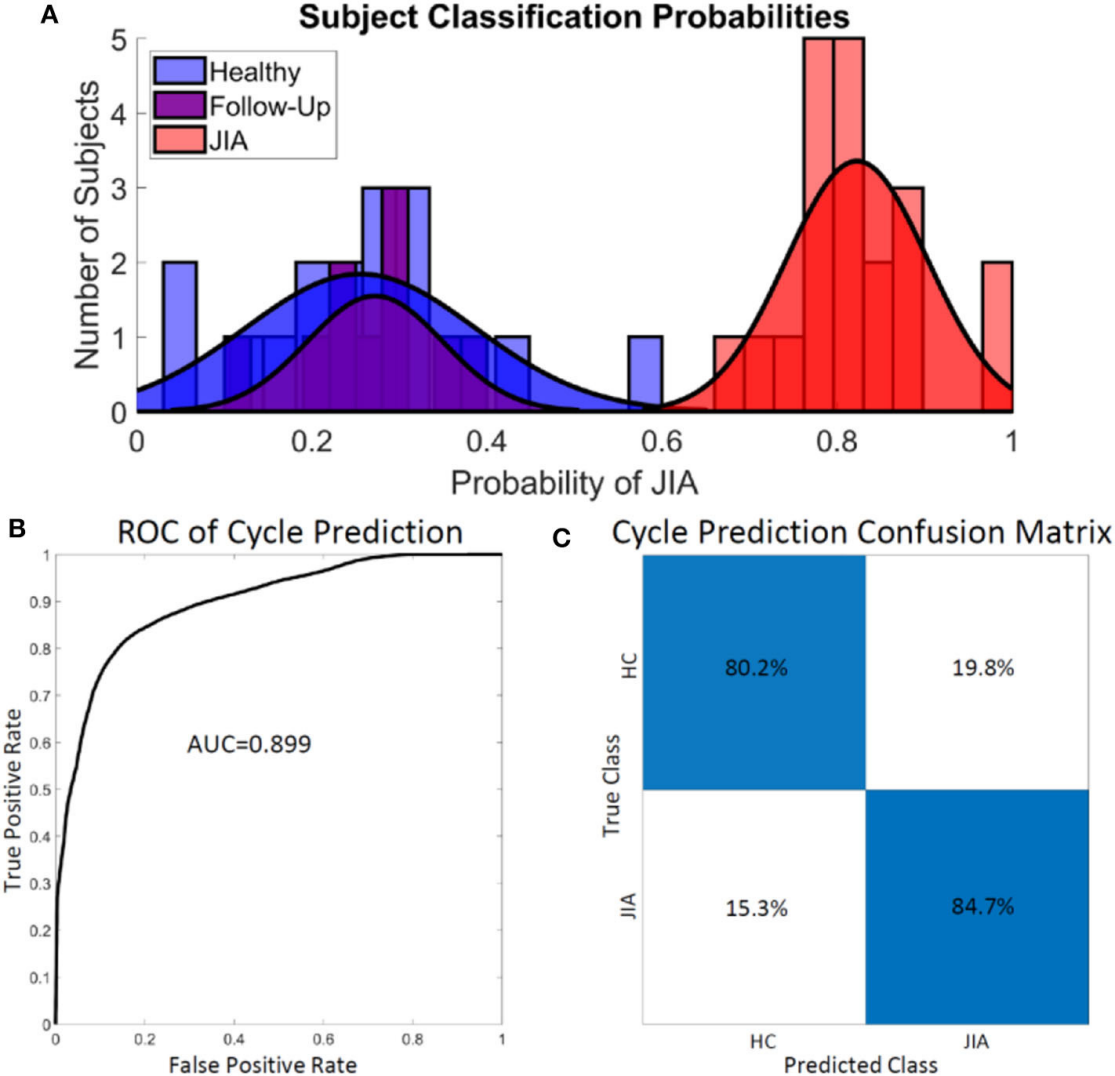
Assessing the performance of the logistic regression classifier on subjects **(A)** and cycles **(B,C)**. **(A)** There was little overlap in the computed joint health score of the healthy control group and the group with JIA. A sub-group from the JIA group after effective treatment had JIA scores heavily overlapping with the healthy control group at follow-up. **(B,C)** The logistic regression model overall classified the individual cycles accurately 82.7% of the time. The model achieved adequately high sensitivity (84.5%) and specificity (80.4%). HC, healthy control.

The knee audio score for each subject was defined as the probability of a cycle belonging to a subject with JIA. In this manner, a knee score of 0 indicates 0 probability of having JIA, and a score of 1 indicates an actively inflamed joint with JIA. A threshold was set at a score of 0.5 to delineate the classification of the two groups. A threshold cutoff of 0.5 was chosen heuristically but could theoretically be changed to place an emphasis on sensitivity vs. specificity as desired. Subjects' joint scores were calculated by averaging all the computed cycle probabilities of each individual subject's flexion/extension cycles. The subject-level joint scores are presented as a histogram in Figure 3A. Notice the heavy overlap between the healthy (blue) and post-treatment, follow-up subjects (purple). This was expected based on the success of the treatment as reported by the treating pediatric rheumatologist. The JIA distribution is centered around a score of 0.82 with clean separation from the other two distributions. The overall cycle-based logistic regression analysis had an accuracy of 82.7% for classifying individual cycles. The receiver operating characteristic (ROC) curve and confusion matrix are presented in Figures 3B,C. The ROC curve had an area under the curve (AUC) of 0.899. The cycle classification had a specificity of 80.4%, a sensitivity of 84.5%, an error rate of 20.1%, a positive predictive value (PPV) of 84.7%, and a negative predictive value (NPV) of 90.2%.

### Feature Importance Ranking and Model Performance

**Figure 4:**
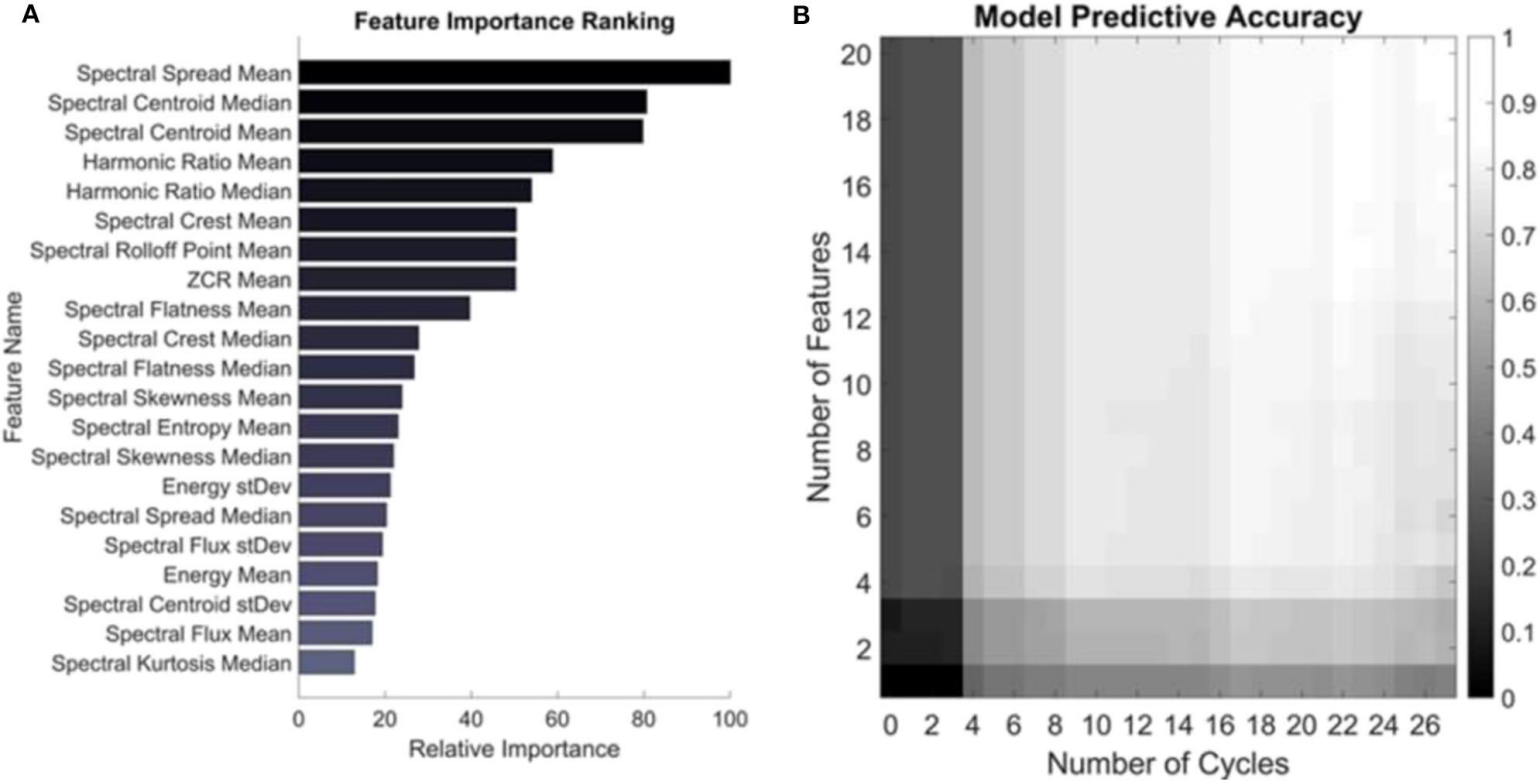
Feature importance and model performance based on number of features and cycles. **(A)** Features are ranked based on their weighted coefficients as output by the trained logistic regression model. The most important feature was the mean spectral spread. **(B)** The model was trained on a feature set containing just one and up to 20 of the top features and the accuracy was assessed based on including those features and number of cycles recorded from a subject. The colors represent the average accuracy across all subjects for all permutations of cycle selection for a given set of testing parameters. The maximum accuracy of 80.6% is seen in the top right corner when trained on the 20 most important features and tested on all cycles of a given subject.

Logistic regression is a binary classification algorithm that finds the best hyperplane in the feature space which separates the two classes: healthy and JIA (20). The absolute values of the individual feature weights describing that hyperplane are used to quantify the impact that each feature has on the model and thus its importance. Figure 4A shows the relative importance of the top 20 features used in computing the knee health score. Of note, the majority of these features for classifying the two classes are in the spectral domain which agrees with the results from our earlier pilot work on the topic (12).

Next, the number of features and cycles were varied to quantify the change in accuracy that the inclusion of each consecutively less important feature and each recorded cycle had on the classification accuracy of each subject. The output of this testing is visualized as an accuracy heatmap in Figure 4B where the color represents the average accuracy from testing on each subject in the dataset using LOSO-CV using the depicted number of features and cycles of movement. At the bottom left of this plot is the accuracy of the model when only trained on the most important feature—the mean spectral spread—and tested on just one randomly selected cycle of flexion/extension from the subject. All permutations of possible cycle selection were performed and averaged to yield the accuracy under these conditions. In the case of just one cycle and one feature, the average cycle classification accuracy was only 11.1%. Ascending along the y-axis, one feature is consecutively added based on its relative importance, such that at the top left corner of the heatmap the model has been trained on the top 20 most important features. Still, when tested with only one cycle from a subject, the accuracy remains low at 25.0%. From left to right, the algorithm is tested on an increasing number of cycles recorded from a subject. The model has an accuracy of 42.8% in the bottom right corner, where it was trained on just the mean spectral spread and tested using all recorded cycles of a subject from all four microphones. The algorithm had the highest accuracy of 80.6% when trained on the top 20 most important features and tested using all recorded cycles. This is slightly <82.7% observed in Figure 3. This discrepancy is because the model in Figure 3 had the added benefit to the classification of all 49 features (Supplementary Table 1), not only the top 20 most important (21).

### Knee Audio Score's Longitudinal Health Tracking Capability

**Figure 5:**
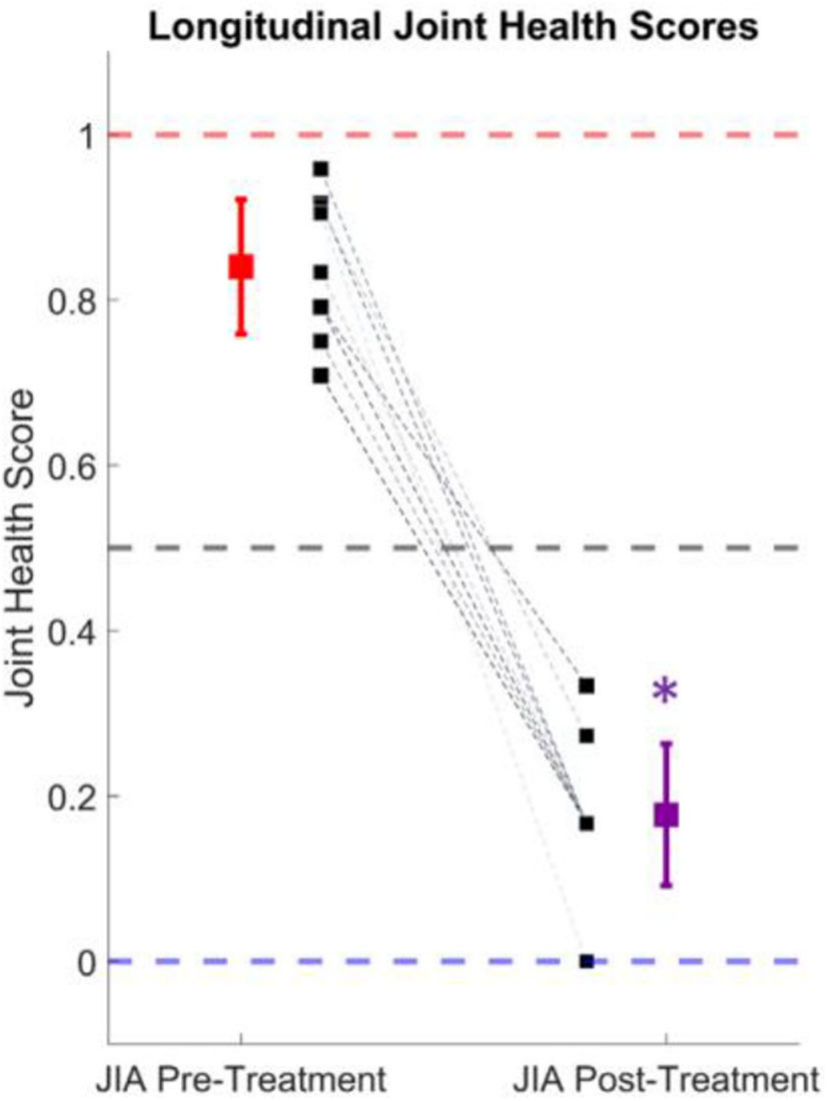
Longitudinal joint health score tracking. The average joint health score, which describes the probability of having JIA, dropped from 0.84 ± 0.08 to 0.19 ± 0.09 after successful treatment of the condition in 10 subjects. The individual subject scores are denoted by the black squares and dashed lines. The mean and standard deviation of the actively inflamed subjects with JIA is shown in red, and the purple marker indicates the mean and standard deviation at follow-up. This drop in joint health score was statistically significant (*p* < 0.001).

The knee audio scores were calculated for 10 of the subjects with JIA before and after 3–6 months of treatment. At first visit, these subjects were either newly diagnosed with JIA, or having a resurgent flare of arthritis. Their treatments were prescribed according to the current clinical standards by their treating pediatric rheumatologist and were recorded but not controlled for in this study. Every subject at follow-up reported a reduction in symptoms and the treating physician reported an overall improvement of the arthritis. In Figure 5, the calculated joint health scores are shown before and after treatment for this cohort. The average joint health score at initial visit was 0.84 ± 0.08. At follow-up, the scores dropped to an average of 0.19 ± 0.09. This drop in joint health scores is statistically significant with a *p*-value = 5.3 × 10^−8^ (tstat = 16.4, 9° of freedom), when tested with a one-tailed *t*-test. The scores from individual subjects are represented with dashed lines in Figure 5 and in all cases mirror the clinical assessment of their improvement.

## Discussion

There is a compelling need for the development of a non-invasively measurable biomarker that can both diagnose and track the status of affected joints in JIA. JIA is a chronic, autoimmune disease of childhood with a highly variable presentation, an etiology linked to genetics and environment, and a complex treatment strategy (9). Assuming a child is properly diagnosed, determining which treatment regimen will work best for them is largely reactive. A certain course of treatment is prescribed and adjusted based on patient-reported feedback and infrequent clinical assessments. In this work, we explore the impact that JAE monitoring could have on the diagnosis and treatment of JIA. If JAEs were found to contain clinically relevant information, they could potentially be used as an initial screening tool by primary care medical professionals – reducing the burden on the healthcare system of unnecessary referrals to specialists. Furthermore, this could help diagnose patients earlier, which may prevent the long-term sequelae of JIA (17). After diagnosis, if joint sounds were found to closely track with treatment efficacy and joint health longitudinally, they could be used as an objective biomarker to decide or even predict the most effective course of treatment. This would reduce the burden of frequent JIA flare-ups on patients and allow for a tightening of the treatment feedback loop leading to overall better management of the condition.

In this study, the effects of JIA on the JAEs produced by articulation of the knee were explored. The study population was made up of 43 subjects, 25 of whom had JIA, and 10 of these 25 subjects had repeat recordings 6 weeks after the initial visit. The JAEs from a pediatric population with JIA of this size have never before been compiled and analyzed. These JAEs were first compared qualitatively to better visualize the differences in the recordings as seen in Supplementary Table 1. It was noted that there are characteristic high frequency clicks in the JAEs of subjects with JIA, that fade away with successful treatment and are not present in matched healthy controls' JAEs. More work is needed to determine the precise mechanistic origin of these high frequency clicks, but we hypothesized that they occur due to increased internal friction in the joint, caused by the characteristic inflammation of the synovial membrane, breakdown of cartilage, and reduced joint space in JIA (3, 22). Of note, similar clicks are apparent in the case of acute injury as was recently discovered by our work in a cadaver model of knee injury (19) and a similar study in an injured athlete model (23). Rather than relying strictly on one or even a few characteristics of these JAEs as was done in previous work, in this study we attempt to more thoroughly quantify the differences between the recorded JAEs. We do this by splitting the joint sound recordings from each subject into their component flexion/extension cycles. On each cycle, 49 features (from the spectral and time-domain) were calculated to describe the observed JAEs. These features and cycles were organized into a feature matrix which was used to train a machine learning, classification model using logistic regression. This technique should provide a more exhaustive analysis of the features of the JAEs, and overall be more generalizable than past efforts to interpret JAEs. The results of this model are described below.

### Knee Audio Score Classification

Logistic Regression and linear discriminant analysis are two of the most widely used statistical methods for analyzing categorical outcome variables. Because logistic regression is more flexible and robust than linear discriminant analysis when considering the assumptions made about underlying data, it is commonly used in medical data binary classification tasks (24). When compared against more complex machine learning models, the modeling parameters in logistic regression are generally easier to interpret rather than a “black-box” approach. This flexibility, robustness, and interpretability should encourage more widespread acceptance of the conclusions provided in this work by the medical research community (20). Logistic regression is a binary classification algorithm that attempts to find the best hyperplane in k-dimensional space for separating the two classes (e.g., healthy and JIA), while minimizing logistic loss (20).

In our application, the logistic regression outputs the probability that a given test cycle belongs to the healthy or JIA class. We have also proposed that the output JIA probability could be used as a basis for quantifying knee joint health. In this paradigm, a probability of 0 indicates a healthy knee with no signs of JIA, whereas a score of 1 indicates a knee clearly affected with JIA. The classification accuracy of the model is presented in *B*. First, the subject-level classification histogram showed clear separation of the joint health scores when the 0.5 classification threshold was applied to the output probabilities (Figure 3A). This finding helps support the idea that knee JAEs could be used as part of the screening and diagnosis of JIA. The accuracy of labeling each cycle is then quantified to better understand the performance of the logistic regression model (Figures 3B,C). The overall accuracy of the cycle labeling was 82.7%, which corresponds to a sensitivity of 84.5% and a specificity of 80.4%. As discussed, JIA is challenging to diagnose not only due to the highly variable nature of the condition and presentation, but also because of the shortage of pediatric rheumatologists who are specially trained to identify the disease. One potential use of JAE-based assessment in JIA is to allow for better screening of the condition by healthcare providers that are less trained to identify it. JAE based assessment is entirely non-invasive and achievable with affordable hardware. The high sensitivity of this technique means that few false positive test results will occur. The technique may be slow to be adopted for final diagnosis, but in the near-future JAEs could at least be used as a preliminary screening tool that gates whether a patient should pursue a specialist consult for further diagnostic workup (i.e., point-of-care screening).

### Feature Importance Ranking and Model Performance

To understand the effects of feature selection and length of recording on JIA JAE assessment, we presented our findings on which signal features are most important for the algorithm, and how it performs with less cycles to classify using a subset of features. In our model, there were 49 features describing each cycle of movement from each subject. A feature weights vector of length 49 was output from the model describing the hyperplane that best separates the JIA from healthy labeled cycles. The absolute values of the individual feature weights were used to quantify the importance of a given feature for the model. The relative importance of the top 20 features in the algorithm are presented in Figure 4A. Each subject had two microphones on each of their knees recording the JAEs during 10 cycles of flexion/extension at a rate of 1 cycle every 4 s. These four audio files are subdivided into the individual cycles of movement based on the simultaneously recorded motion data captured by the inertial measurement unit (IMU) attached to the subjects' ankles. The resulting data structure thus had approximately 40 segments of data describing one subject's movement. Figure 4 graphically depicts the results of varying the number of those segments included in the testing dataset. Each square in Figure 4 describes the average accuracy when each subject was tested with the described parameters as a part of LOSO-CV on the trained model. Along the y-axis, features were sequentially added in order of descending importance, such that at the bottom of the plot, only the most important feature—the mean spectral spread—was used to classify the cycles. Upon ascending the y-axis, each of the 20 features as described in Figure 4A are consecutively included in training the logistic regression model. This figure thus depicts the impact that feature selection has on the accuracy of the classification. There is a clear benefit on the accuracy of the model by including more features, and this should help with the generalization of the model to novel data. In the past, attempts have been made to describe knee JAEs using only one or a few different signal features (14, 25, 26). These attempts generally have success on a small data set, but when applied to a data set of this size were suboptimal when compared to the accuracy of the model proposed in this work.

The impact of the length of the JAE recording is also demonstrated in Figure 4B from left to right. Each step to the right includes an additional, and randomly selected, flexion-extension cycle, and the color of the square indicates the accuracy of classifying a subject with that many cycles. On the left, we test the model with only one cycle recorded from one microphone on each subject. On the far right, every cycle recorded for every microphone is used to test any given subject. The impact is similar to increasing the number of features in the trained model – as the number of cycles increases the classification accuracy similarly increases. Note that there is some possible redundancy in having two microphones recording the JAEs from each knee. In this case, the impact of having similar recordings in two of the microphones can be noted by the relative plateau of the accuracies around the 18th recorded cycle (accuracy is no longer substantially increasing with each added cycle). Overall, this analysis demonstrates the impact that the feature selection and length of JAE recording has on the accuracy of the model. In our case, the accuracy was at its lowest with one feature and one cycle at 11.1% and achieved a high of 80.6% with the top 20 most important features and every recorded cycle from a subject. This analysis also demonstrates why past approaches have had only limited success in generalizing their findings. If only a subset of these features were used to describe JAEs, the accuracy would significantly diminish. Many features are needed to fully describe the nature of these sounds and separate the differences between populations. Later work comparing a different clinical scenario, or a larger dataset may find that a different feature is more important for delineating two study groups, but the approach applied in this paper should hopefully provide guiding influence on future assessments of JAEs.

### Longitudinal Joint Health Tracking

To discover if knee JAEs had the potential for quantifying joint health longitudinally, 10 subjects with JIA had their JAEs recorded during an active flare-up of the condition and 3–6 months later at their follow-up visit. In this particular cohort, every subject showed clinical improvement and reported a lessening of symptoms. To calculate these subjects' knee scores, the logistic regression model was trained on all subjects not in this cohort. The recordings before and after treatment were tested on the trained model and the knee audio scores computed as described in the section “Knee Audio Score Classification Using Logistic Regression”. The hypothesis was that as a child's knees healed from effective treatment, their knee scores would decrease from the JIA range (0.5–1.0) toward the healthy range (0.0–0.5). In all subjects, this hypothesis was shown to be valid. There was a statistically significant drop in the average scores of 0.65, or a 77.4% improvement in the joint health score. This closely tracked with the reported clinical workup of the subjects indicating that joint health scores based on JAEs may be clinically applicable for not only diagnosing JIA (as discussed in section Knee audio score classification), but also monitoring the condition over time.

In this study, these 10 patients represent a subset of the overall JIA population and before claiming how consistently joint sounds track with knee health status in an individual the sample size of those studied should be further increased. However, these findings represent the first time that a population large enough to adequately power a study of children with JIA has been assessed longitudinally. The close correlation between the change in joint sounds and the observed clinical status supports further research into this relationship. Overall, this study represents an early, but important step toward understanding the nature of JAEs. The strong separation of the classes alongside the close tracking of disease activity make it clear that JAEs contain clinically relevant information. This information if properly leveraged could 1 day enable better more personalized treatment of JIA.

### Limitations and Steps to Clinical Adoption

JIA is a chronic condition that affects multiple joints in the body. The knee is one of the most commonly affected joints and made for a viable target for this attempt at analyzing JAEs. To better understand the clinical utility of this sensing modality, JAEs should be studied in other commonly affected joints in JIA. Additionally, the sensitivity of this method should be compared against the performance of the current clinical standard procedure for diagnosing and staging the condition, as well as against other modalities such as magnetic resonance imaging or ultrasound, which typically are time consuming and expensive. Treatment of JIA seeks to reduce the frequency of acute, symptomatic flare-ups, and to ultimately achieve clinical remission. In this study, the treatments our subjects underwent were not controlled for due to the small sample size. In the future, the effectiveness of therapy should be quantified using a prospective study design. Additionally, in this cohort all subjects improved with treatment and we observed a corresponding drop in the joint health score. Since no patients got worse at follow-up, we were unable to discover if JAE assessment could track worsening of the condition. The sensitivity of joint sounds for detecting not only different severities of the condition but also the course of the condition should also be assessed. JAEs would be significant clinically if they were able to determine the difference between an acutely inflamed joint and a more chronic, undiagnosed state. Determining that duration of disease activity would help with selecting the ideal treatment for a patient. Classifying subjects into the different subtypes of JIA and delineating joint sounds caused by JIA vs. all other causes would also offer clinical merit. This study was performed on a fairly large sample size of subjects to date, and enrollment is ongoing to support future work. Increasing the number of subjects would better support the generalizability as well as mitigate possible overfitting of the discussed results. Overall, in this paper we present JAEs as a novel technique for analyzing the health of a joint in JIA. The findings in this paper present significant clinical merit to this type of analysis, but there is still much to be discovered.

## Materials and Methods

### Human Subject Protocol and Subject Demographics

The study was conducted under a protocol approved by the Georgia Institute of Technology and Emory University Institutional Review Boards. Forty-three subjects participated in this study after completing a written informed consent. Twenty-five of the subjects were diagnosed with JIA by a pediatric rheumatologist and 18 of the subjects were healthy controls with no history of JIA or acute knee injuries. The group with JIA consisted of 20 females and five males (12.2 ± 3.1 years old, BMI 20.1 ± 4.1 kg/m^2^). The healthy control group consisted of 15 females and three males (12.9 ± 2.7 years old, BMI 22.3 ± 2.8 kg/m^2^) with no history of joint disease, surgery or significant joint injury. To capture longitudinal changes in the knee JAEs during the course of treatment, data were acquired from 10 of the subjects (1 male, 9 female, 12.5 ± 3.3 years old, BMI 20.8 ± 3.5 kg/m^2^) with JIA a second time, 3–6 months after initial measurements (follow-up group). Note, that JIA is more prevalent in females with estimates ranging from 65–78% of all cases occurring in females, thus the demographics of this study were selected accordingly to match this distribution as closely as possible (27, 28).

The data acquisition set up for each subject is shown in Figure 1B. To record the sounds produced by the joints, two uniaxial analog accelerometers (3225F7, Dytran Instruments Inc. Chatsworth, CA) were attached 2 cm medial and lateral to the distal patellar tendon using double-sided adhesive pads (Rycote Microphone Windshields Ltd, Stroud, Gloucestershire, GL5 1RN, United Kingdom) on both knees. These professional-grade pads tightly coupled the accelerometer to the subject's knee. This accelerometer has a broad bandwidth (2 Hz−10 kHz), high sensitivity (100 mV/g), low noise floor (700 μgrms), miniature size and low weight (1 gram). This accelerometer placement location has been shown to allow for the of capture high-fidelity signals capable of differentiating meniscus injury status in an JAE cadaver model (19).

To record the knee JAEs, each subject performed 10 unloaded knee flexion/extension exercises, while seated on a height-adjustable stool to prevent foot contact with the ground. The subjects repeated the movement as seen on an instructional cartoon that encouraged a cycle to be completed every 4 s through the full range of motion (RoM) of each subject. The signals from the accelerometer were sampled at 100 kHz and recorded using a data acquisition module (USB-4432, National Instruments Corporation, Austin, TX). An inertial measurement unit (IMU) attached around the ankle of the subject recorded synchronous positional data during JAE recording at 50 Hz to allow for analysis on a cycle-by-cycle basis, as well as to ensure the subject maintained an appropriate speed and RoM. The ideal speed and angles to move through have previously been explored using a cadaver model of JAEs (19). The exercise and recording protocol were repeated for both knees for all subjects. The recorded signals were analyzed using Matlab (MathWorks, Natick, MA).

### Signal Processing and Feature Extraction

The JAEs were analyzed in the time and frequency domains. Figure 2 shows a representative plot of the time domain signal after bandpass filtering from one subject with JIA, that subject's JAEs at their 3-months follow-up visit, and a healthy, matched control's JAE recording. It is notable that the number of spikes in the time domain of the patient with JIA went down with effective treatment as seen at follow-up to more closely resemble the JAE recording from the healthy control. The JAEs from these subjects have high bandwidth frequency content as expected from earlier pilot work (25, 29, 30). Figure 1C graphically depicts the signal analysis workflow for knee JAEs. The signals are pre-processed using a digital finite impulse response (FIR) band-pass filter with 250 Hz−10 kHz bandwidth. The bandwidth employed in this filtering is based on prior work: at the low end, the cutoff of 250 Hz is selected to reduce low frequency artifacts and muscle sounds (<100 Hz) while preserving the sub kHz friction-generated components of the sounds; at the high end, the cutoff of 10 kHz is selected to remove high frequency artifacts while still preserving the kHz range of frequencies responsible for the acoustic emissions that are observed from the joint. To segment the JAE data into individual flexion/extension cycles, an FIR low-pass filter (5 Hz) is applied to the raw JAE signals to visualize the movement of the knee through its RoM. This motion data is compared against the synchronized IMU data and the proper indices for the beginning and end of each flexion/extension cycle were selected. These individual cycles were separated and subdivided into 400 ms long frames. This frame (or window) length was selected to provide sufficient width to capture lower frequency information while still providing multiple frames per flexion/extension cycle. A total of 49 signal features are extracted from each frame for each microphone, comprising features that—in our group's prior work, and in audio processing and classification work in other domains—have been found to contain salient information. Feature descriptions are available in Supplementary Table 1. The 10 frames corresponding to one cycle are averaged to give 49 descriptors of each cycle of flexion/extension. This process was repeated for all four microphones – two on each knee. These feature sets were stored in the row-matrix, ***X***. The rows of ***X*** each represent a single cycle of movement as recorded from each microphone, and the columns represent each of the 49 features extracted. The matrix ***X*** was standardized to zero mean and unit variance by subtracting the mean of each column and dividing by its standard deviation (see Feature Matrix in Figure 1C).

The features extracted can be categorized into two groups: either time domain or spectral features. The time domain features include the zero-crossing rate (ZCR), energy, root-mean-square (RMS) amplitude, and entropy. The frequency characteristics of the joint sounds are described by the spectral features including the spectral centroid, spectral flux, spectral density, spectral roll-off, spectral spread, and spectral entropy (A full list of the features is available in Supplementary Table 1.)The mean, standard deviation, and coefficient of variance are all computed for the set of 400 ms windows on each cycle to better classify these features. This approach using these particular features to classify joint JAEs is based on the appearance and sound of the signals, and their selection was supported by previous pilot work on this topic (12, 31).

### Knee Audio Score Classification Using Logistic Regression

With the data appropriately organized, we trained a logistic regression classification model. Logistic regression is a common statistical machine learning technique for binary classification problems (e.g., healthy vs. JIA). At the core of this algorithm is the logistic function, which was originally developed by ecologists to describe population growth – it is a sigmoidal curve that rises quickly and levels off at a given environment's carrying capacity (32, 33). The algorithm uses this function to map any real number input to a value between 0 and 1.


(1) $$\begin{array}{r} \frac{1}{\left(1+e^{-1}\right)} \end{array}$$

#### Logistic Function

In logistic regression, the input values (*x*_1_ …* x*_*n*_) are combined linearly to predict an output value (*y*) using weighted coefficients (*b*_0_ …* b*_*n*_). However, unlike linear regression, in logistic regression the output values being predicted are binary (0 or 1, or in our case healthy or JIA). The logistic regression equation thus takes on the following format:


(2) $$\begin{array}{r} y=\frac{e^{b_{0}+b_{1}x_{1}+\ldots +b_{n}x_{n}}}{1+\;e^{\left(b_{0}+b_{1}x_{1}+\ldots +b_{n}x_{n}\right)}} \end{array}$$

#### Logistic Regression Mapping Function

Where y is the predicted output, *b*_0_ is the intercept, *b*_1_ – *b*_*n*_ are the coefficients for the input feature values (*x*_1_ – *x*_*n*_). In our use case, *x* corresponds to a row of matrix ***X***, which contains the values of each of the 49 computed signal features for an individual cycle from one accelerometer. Once trained, each column of the input matrix ***X*** (i.e., each feature) has an associated coefficient learned through training (*b*_1_ – *b*_*n*_). The vector of *b*_1_ – *b*_*n*_ is stored in the coefficient vector (β). β is found using a maximum-likelihood estimation (MLE), specifically the quasi-Newton method, that minimizes the error of the predicted probabilities (20, 34, 35).

The predicted output (*y* in **Equation 2**) is the probability that a given input belongs to the default class, selected in our case as JIA. These probability predictions are transformed into binary values (0 or 1) in order to create the final probability-based predicted label for each feature using the threshold in **Equation 3**.


(3) $$\begin{array}{l} If\;mean\left(p\left(\text{x}\right)\right)\;\leq \;0.5,\;y\;=\;Healthy\\ If\;mean\left(p\left(\text{x}\right)\right)\;>\;0.5,\;y\;=\;JIA \end{array}$$

#### Threshold for Healthy Control vs. JIA Classification

As mentioned, each cycle of flexing/extension (each row of ***X***) is classified on as a 0 or 1, with 0 representing a healthy, unaffected knee and 1 representing a knee with active JIA. To calculate this score, we train a logistic regression classification model. All rows for a subject can be removed from ***X*** to leave behind ***X'*** and **X**_subject_. Each row in these matrices corresponded to one accelerometer's output for one cycle of movement. Each subject had two accelerometers on each leg and was asked to perform 10 cycles of flexion/extension. The average number of rows in these submatrices was 36 ± 3 rows, with the average number of rows per accelerometer being 8 ± 1 rows. If the majority of the predicted labels for an individual row were classified as 0, the cycle was labeled as healthy. If the majority of the predicted labels were predicted as 1, the cycle was considered to be JIA. In this way, the median of the predicted labels of each row determines the classification of that cycle of that microphone. The median of the rows in any given ***X***_subject_is taken to be the subject classification. If the majority of the rows was predicted to be 1's, the subject was labeled as having JIA. Inversely, if the majority of rows was predicted as 0's, the subject was labeled as healthy.

The logistic regression model's performance was assessed using LOSO-CV (36). In each fold of this validation, the logistic regression classifier was trained using the data in ***X'*** with one subject omitted - ***X***_subject_. The trained model then classified the signal of the excluded subject's knee JAEs. During LOSO-CV, the matrix ***X'*** was standardized after the removal of ***X***_subject_. The mean and standard deviation of ***X'*** were then subtracted and divided, respectively, from the columns in ***X***_subject_. By doing this, the calculated features for ***X***_subject_ were not prematurely included in the standardization of ***X***. The model estimates the probability of JIA for each row (cycle) in ***X***_subject_. These probabilities were stored in the vector, ***p***_***predicted***_. The overall subject's audio scores were calculated by averaging the contents of ***p***_***predicted***_ (Figure 1D). The 0.5 threshold was applied to this average probability to assign the predicted label of healthy (0) or JIA (1). The cross-validation was completed by calculating knee audio scores for all 43 subjects, excluding one subject per fold. The follow-up recordings were not included in this model accuracy calculation because the treating physician stated they were along a spectrum of convalescence, and thus their ground truth label was unknown. The generalizability of the model is assessed by calculating the accuracy of our algorithm in labeling each cycle, as well as in labeling each.

The average probabilities were used not only for predicting labels, but also as an indicator of knee health. In this way, as the average probability of a subject trends toward 0, the signal more greatly resembles a healthy knee. For subjects with JIA that have follow-up recordings, this process was repeated to calculate the change in the probability of JIA between the first recording and second. Importantly, the follow-up recordings are never used as part of the training set, since at the time of recording those subjects the ground-truth of their disease status is unknown.

### Feature Importance Ranking

The relative weighting of each of the features in the model needs to be explored to understand which features most relate to differentiating JAEs from patients with JIA compared to healthy controls. To quantify the importance of each feature, the standardized data from every subject with JIA (excluding the follow-up data due to it lacking a ground truth classification) is used to train the classifier. The resulting model is used to generate relative feature importance scores. In this case, no testing set is required to quantify feature importance since we are not assessing the generalizability of the model. In the case of logistic regression, the model computes a coefficient for each input feature that describes the *k-*dimensional hyperplane that best separates the two input classes. When the input matrix ***X*** is standardized to zero mean and unity variance, the absolute value of each of the coefficients output from the model can be directly compared to assess relative importance to the model. In this way, a coefficient with a large absolute value has a larger effect on the model than one with a smaller absolute value. All 49 features are ranked in order from most to least important as seen in Figure 4A.

### Effect of Number of Features and Cycles of Movement on Model Performance

After ranking the 49 features, we further assessed the impact on the accuracy of the model's predictive capabilities by training the model on one to forty-nine features in order of their relative importance. We first trained a model on only the most important feature, and assessed the accuracy of the model as detailed above using LOSO-CV. Next, we iteratively added each new feature in order of descending relative importance to observe how that accuracy improved with the addition of each new feature. We simultaneously assessed the importance of the number of flexion/extension cycles by testing each iteration of the model on a subset of all of the cycles. For example, we first trained the model on the most important feature, and tested the model using one cycle from the subject left out, next two cycles, then three cycles, all the way up to the full number of recorded cycles. In doing so, we calculated how the model responded for each feature input and for each additional cycle of movement input. Of note, when choosing the subsets of cycles to test we iteratively tested up to 1,000 unique permutations on any given sized subset of cycles and the average of those cycles was reported. A heatmap of these results was generated and can be seen in Figure 4B.

## Data Availability Statement

The raw data supporting the conclusions of this article will be made available by the authors, without undue reservation.

## Ethics Statement

The studies involving human participants were reviewed and approved by Emory University School of Medicine Institutional Review Board Georgia Institute of Technology Institutional Review Board. Written informed consent to participate in this study was provided by the participants' legal guardian/next of kin.

## Author Contributions

DW served as the project lead and was involved in every part of its design, execution, analysis, and reporting. JZ provided his machine learning expertise and helped design the JAE algorithm. SG helped organize the data and performed the IMU assessment. TG and LP were lead clinical coordinators that helped devise an appropriate protocol for consenting, assenting, and recording JAEs in a clinical setting. OI served as the principal investigator for the project, and was integral in the funding, managing, planning, and execution of all aspects. SP closely collaborated with OI and sponsored this project: specifically, SP provided access to patients, clinic space, and medical expertise on the current state of JIA management. All authors contributed to the article and approved the submitted version.

## Conflict of Interest

The authors declare that the research was conducted in the absence of any commercial or financial relationships that could be construed as a potential conflict of interest.
