# Supplementary material for: Knee Acoustic Emissions as a Digital Biomarker of Disease Status in Juvenile Idiopathic Arthritis
Source: Front Digit Health. 2020 Nov 19;2:571839. doi: 10.3389/fdgth.2020.571839 (PMC8521909; doi:10.3389/fdgth.2020.571839)
Supplement: Supplementary file 1 [file Table_1.docx]

**Supplementary Materials:**

*Table S1. Features used in logistic regression model.* Each feature had there mean, median, and standard deviation used in the model classifier as appropriate. Descriptions found at ^30,36^.

| # | Feature Name | Description |
| --- | --- | --- |
| 1 | Mean Frequency | Center of the power distribution across all frequencies of a signal. |
| 2 | RMS Amplitude | Root mean square (RMS) of the signal amplitudes. |
| 3 | Zero Crossing Rate | The rate of sign-changes of the signal. |
| 4 | Energy | Square of the time-domain signal amplitudes, corresponds to loudness of signal. |
| 5 | Fundamental Frequency | Pitch of a signal - for a harmonic signal it’s the frequency such that its integer multiple best explains the content of the signal spectrum. |
| 6 | Spectral Centroid | The barycenter, or "center of gravity", of the frequency spectrum |
| 7 | Spectral Spread | Standard deviation around the spectral centroid. |
| 8 | Spectral Crest | Ratio of the maximum of the spectrum to the arithmetic mean of the spectrum , indicates peakiness of spectrum. |
| 9 | Spectral Decrease | Represents the amount of decrease of the spectral amplitude – related to perceptual studies. |
| 10 | Spectral Entropy | Measures the disorder and peakiness of the spectrum |
| 11 | Spectral Flatness | Measures the noisiness to sinusoidality of a spectrum. |
| 12 | Spectral Flux | Measures the variability of the spectrum over time |
| 13 | Spectral Kurtosis | Measures the flatness, or non-Gaussianity, of the spectrum around its centroid. |
| 14 | Spectral Roll off Point | Measures the frequency so that 95% of the signal energy is contained below that frequency |
| 15 | Spectral Skewness | Measures of the asymmetry of the frequencies around the centroid |
| 16 | Spectral Slope | Measures the amount of decrease of the spectral amplitude. |
| 17 | Harmonic Ratio | Measures the amount of energy in the tonal part of the signal compared to the total energy. |
